# Real-life use of onabotulinumtoxinA for symptom relief in patients with chronic migraine: REPOSE study methodology and baseline data

**DOI:** 10.1186/s10194-017-0802-6

**Published:** 2017-09-06

**Authors:** Brendan Davies, Charly Gaul, Paolo Martelletti, Juan Carlos García-Moncó, Stephanie Brown

**Affiliations:** 1 Royal Stoke University Hospital, Newcastle Road, Stoke-on-Trent, Staffordshire ST4 6QG UK; 2 Migraine and Headache Clinic Königstein, Königstein im Taunus, Germany; 3 Regional Referral Headache Centre, Sapienza University, Rome, Italy; 4 Hospital de Galdacano, Vizcaya, Bilbao, Spain; 5 Allergan plc, Marlow, Buckinghamshire UK

## Abstract

**Background:**

Migraine is a debilitating neurological disorder that affects 14.1% of the US and 14.7% of the European populations. Chronic migraine (CM) is broadly defined as headache occurring on ≥15 days per month for ≥3 months, and has an estimated worldwide prevalence of 1.4% to 2.2%. OnabotulinumtoxinA is currently approved for the treatment of CM in most European countries, and is the only *preventative* treatment approved for adults with CM, based on results from the PREEMPT clinical trial programme. The ongoing prospective, observational REal-life use of botulinum toxin for the symptomatic treatment of adults with chronic migraine, measuring healthcare resource utilisation, and Patient-reported OutcomeS observed in practice (REPOSE) Study aims to describe real-world healthcare resource utilisation and patient-reported outcomes over a 2-year period in Germany, Italy, Norway, Russia, Spain, Sweden, and the United Kingdom, among patients with CM prescribed onabotulinumtoxinA.

**Methods:**

Herein, methodology and baseline characteristics of patients who participated for ≥6 months in REPOSE are reported. No outcomes data are presented, although the methods for collecting these data are detailed. In REPOSE, onabotulinumtoxinA is administered at baseline and each follow-up visit (approximately every 3 months) during the 24-month treatment period, according to the treating physician’s best clinical judgment and standard of care, guided by the terms of the marketing authorisation described in the Summary of Product Characteristics. Outcome assessments include Migraine-Specific Quality of Life Questionnaire (MSQ), EuroQol Group Questionnaire (EQ-5D), headache-day frequency, treatment satisfaction, headache-related healthcare resource utilisation (ie, healthcare professional visits, hospital admissions, medication use), onabotulinumtoxinA utilisation (ie, dose, sites), and safety/tolerability.

**Results:**

As of the interim assessment date for this analysis, the study has enrolled 644 patients from 78 sites throughout Europe, and baseline data are available for 336 patients from 61 sites who participated in the study for ≥6 months. Baseline measures indicate substantial disease burden and healthcare resource utilisation.

**Conclusions:**

Final results from the REPOSE Study will provide the largest real-world, long-term analysis of the clinical use of onabotulinumtoxinA for the treatment of CM and will add important information to existing real-world findings. Future analyses will assess the long-term safety and efficacy of onabotulinumtoxinA in this population.

## Background

Migraine is a debilitating neurological disorder that affects 14.1% of the US [1, 2] and 14.7% of the European [3] populations. It can be categorised on the basis of headache-day frequency into episodic (<15 headache days/month) and chronic forms. Chronic migraine (CM) is defined by the International Classification of Headache Disorders (third edition, beta version [ICHD-3b]) as headache occurring on ≥15 days per month for >3 months, with ≥8 days per month meeting criteria for migraine with or without aura [4]. Chronic migraine has an estimated worldwide prevalence of 1.4% to 2.2% [5], and is associated with substantial disease burden. Compared with episodic migraine (EM), patients with CM suffer from greater disease impact [6], a higher level of disability [7, 8], higher scores for depression and anxiety [9], and reduced quality of life [7]. In addition, CM is associated with higher healthcare utilisation and reduced productivity, resulting in an overall greater economic burden compared with EM [10, 11, 7]. This economic burden has been reported to be reduced by onabotulinumtoxinA treatment in cases of CM refractory to ≥2 oral prophylactics, with respect to migraine-related emergency department visits, urgent care visits, and hospitalisations [12].

Although several classes of medication are used as preventative treatments for CM (eg, beta blockers, antidepressants, muscle relaxants [eg, tizanidine], anticonvulsants) [13], most (with the exception of topiramate [an anticonvulsant] [14, 15] and amitriptyline [an antidepressant] [16, 17]) do not have empirical evidence of efficacy in CM. Furthermore, of these medicines, only topiramate and beta blockers are approved for migraine prevention (although neither is approved for CM prevention specifically). In addition, adherence to these medications is typically poor [18], largely due to adverse events [19] and possibly also due to lack of sustained efficacy. The lack of safety and efficacy of these agents suggests an unmet need for improved preventative treatment of CM [19, 20].

OnabotulinumtoxinA has received regulatory approval in most European countries as the only approved treatment for symptom relief in adults with CM [21]. Recommended treatment consists of intramuscular injections of 155 U divided among 7 head/neck muscles administered in ~12-week intervals [22]. The Phase 3 REsearch Evaluating Migraine Prophylaxis Therapy (PREEMPT) clinical trials evaluated the safety and efficacy of onabotulinumtoxinA for the preventative treatment of CM. Compared with placebo, patients treated with 2 cycles of onabotulinumtoxinA (ie, 24 weeks) experienced a significant reduction in headache days (per 28-day period) and an improvement in quality of life [23, 24]. In a pooled analysis of those patients who continued treatment for an additional 3 cycles (ie, 52 weeks), a persistent improvement in the number of headache days and quality of life was observed with onabotulinumtoxinA [25]. Throughout the 52-week study period, onabotulinumtoxinA was generally well-tolerated, and the adverse event (AE) profile was consistent with previous reports.

The PREEMPT clinical trial programme provides support for the use of onabotulinumtoxinA for the preventative treatment of adults with CM over a 1-year period, and several subsequent studies have supported these findings in patients with and without medication overuse [26–33]. The ongoing prospective, observational REal-life use of botulinum toxin for the symptomatic treatment of adults with chronic migraine, measuring healthcare resource utilisation, and Patient-reported OutcomeS observed in practice (REPOSE) Study aims to describe real-world healthcare resource utilisation and patient-reported outcomes among patients with CM who were treated with onabotulinumtoxinA over a 2-year period. This report will review the REPOSE Study methodology and baseline patient characteristics. No outcomes data are reported here, although the methods for collecting these data are detailed.

## Methods

### Study design

The REPOSE Study is an ongoing 24-month, prospective, non-interventional, observational study being conducted among 78 clinics throughout Germany, Italy, Norway, Russia, Spain, Sweden, and the United Kingdom. Patients prescribed onabotulinumtoxinA (BOTOX®, Allergan plc, Dublin, Ireland) for symptom relief of CM are recruited over a 6- to 12-month period. The 24-month treatment period, including follow-up, is carried out per the treating physician’s best clinical judgment and standard of care, guided by the terms of the marketing authorisation described in the Summary of Product Characteristics [34]. Treatment, therefore, reflects the individual physician’s usual clinical routine and country-specific standards of care. The total study duration is 30 to 36 months and consists of enrollment, a baseline visit (visit 1) and follow-up visits approximately every 3 months. A window of at least 15 days spanned the 3-month follow-up time point, such that 3 slightly different groups could be defined as follows: less than 3 months, <75 days since the last injection; every 3 months, ≥75 and <105 days since the last injection; more than 3 months, ≥105 days since the last injection. OnabotulinumtoxinA injections are administered at baseline and each follow-up visit. All participating clinics obtain appropriate ethics committee approval and the study is being conducted in accordance with International Conference on Harmonisation Good Clinical Practice. All patients provide written informed consent.

### Study treatment

Treating physicians are trained on onabotulinumtoxinA injection patterns as described in the Summary of Product Characteristics [34] and the PREEMPT study paradigm [35]; although there was no mandate for physician’s to comply with these injection paradigms. OnabotulinumtoxinA is administered in 0.1 mL injections (5 U) using a 30-gauge, 0.5-in. needle. The minimum recommended dose of onabotulinumtoxinA is 155 U injected bilaterally into 31 sites among 7 head and neck muscles. At the physician’s discretion, the protocol allows for an additional 8 injections according to the ‘follow-the-pain’ strategy for a total dose of 195 U [35]. Injection sites and recommended doses are outlined in Table 1.

**Table 1:** OnabotulinumtoxinA Injection Sites and Doses [35]

Muscle Injection Site	Minimum Recommended Dose	Additional Injections per the ‘Follow-the-Pain’ Strategy
Frontalis	20 U, 4 sites	NA
Corrugator	10 U, 2 sites	NA
Procerus	5 U, 1 site	NA
Occipitalis	30 U, 6 sites	10 U, 2 sites
Temporalis	40 U, 8 sites	10 U, 2 sites
Trapezius	30 U, 6 sites	20 U, 4 sites
Cervical paraspinal muscle group	20 U, 4 sites	NA

NA = not applicable

The cost of onabotulinumtoxinA and treatments reflected real-world costs, and varied depending on country and insurance status of the patient.

### Patient selection

This report includes patients who participated for ≥6 months based on an interim analysis date of February 12, 2015. Male and female patients aged ≥18 years who are prescribed onabotulinumtoxinA for CM symptom relief are enrolled. Patients are excluded if they had received any botulinum toxin serotype within the previous 26 weeks, if they are currently participating in the Botox Chronic Migraine Post-Authorisation Safety Study (CM PASS), or if they are contraindicated for treatment with onabotulinumtoxinA per the prescribing information. Given the observational nature of this study, no other formal exclusion criteria have been defined. Patients are free to leave the study at any time, independent of their response to treatment. Any pregnancies that occur during the study are reported to Allergan via the electronic case report form (eCRF) within 24 h of confirmation. In addition, the patient’s primary care physician is notified that the patient has been treated with onabotulinumtoxinA. The patient is then withdrawn from the study with a safety follow up of at least 12 weeks and is followed to term by the treating physician. A final pregnancy outcome report is then provided to Allergan.

### Assessments

At the baseline visit, patients are screened for inclusion criteria, provide informed consent, and receive their first treatment with onabotulinumtoxinA. Baseline assessments include demographics (ie, age, gender, height, weight, body mass index [BMI], education and employment status), medical history, and migraine history (ie, diagnosis, age of onset, time since diagnosis of migraine, and time since diagnosis of CM). All efficacy, healthcare resource utilisation, onabotulinumtoxinA utilisation, safety, and tolerability data are assessed at baseline and each follow-up visit as described below.

#### Efficacy

Patient-reported efficacy measures include the Migraine-Specific Quality of Life Questionnaire (MSQ) version 2.1, the EuroQol Group EQ-5D Questionnaire, patient-reported estimation of headache day frequency, and patient-reported treatment satisfaction.

The MSQ version 2.1 is a 14-item quality of life questionnaire that measures the impact of migraine on daily activities. The scale consists of 3 domains: 1) role restriction (questions 1–7) describes the degree which daily activities are limited; 2) role prevention (questions 8–11) describes the degree which daily activities are interrupted; and 3) emotional function (questions 12–14) describes the feelings of helplessness and frustration resulting from migraine. Each item is scored on a 6-point scale (1 = none of the time; 2 = a little bit of the time; 3 = some of the time; 4 = a good bit of the time; 5 = most of the time; 6 = all of the time); dimension and total scores are calculated as the sum of the raw item scores rescaled from 0 to 100, where a higher score indicates a better quality of life [7, 36, 37]. The MSQ is reported as change from baseline in total and dimension scores.

The EuroQol Group EQ-5D questionnaire assigns an overall health state classification, reported as an index score and a patient-reported visual analog scale (VAS) health state score. The index score is calculated on the basis of 5 dimensions: 1) mobility; 2) self-care; 3) usual activities; 4) pain/discomfort; and 5) anxiety/depression, with each dimension rated on a 3-point scale (1 = no problems; 2 = some problems; 3 = extreme problems). Starting with a score of 1.0, weighted scores are deducted on the basis of each dimension’s rating to yield an outcome score ranging from −0.59 to 1.00. A score of 1.00 indicates ‘full health,’ a score of 0 indicates ‘death,’ and a negative score indicates a health state that is perceived to be worse than death [38]. The VAS score is a patient-reported measure of the current health state ranging from 0 (worst health imaginable) to 100 (best imaginable health state). The EQ-5D is reported as change from baseline in the index and health state scores, as well as a frequency distribution of perceived problems for each dimension.

The frequency of headache days is estimated by each patient as the number of days within the past month with ≥4 h of continuous headache.

Satisfaction with onabotulinumtoxinA treatment is assessed by both the patient and the prescribing physician as ‘insufficient,’ ‘moderate,’ ‘good,’ or ‘very good’ and is reported as a frequency distribution of satisfaction level as well as the proportion of patients and physicians who rated satisfaction as ‘good’ or ‘very good.’

#### Healthcare utilisation

The frequency of headache-related healthcare professional (HCP) visits (by type: primary care consultation, outpatient consultation, accident and emergency visit, alternative practitioner, and other) and hospital admissions for headache are recorded for each period between visits. The total number of HCP visits and hospital admissions are normalised to 90 days using the following formula: (90 ÷ number of days between periods) × number of visits to HCPs or admissions to hospitals between periods. The frequency and proportion of patients who had visits or admissions between each visit is compared with those of the 3 months before baseline. At baseline, a complete medication history is documented, including headache medication used at any time before baseline, headache medication or therapy prescribed in the 26 weeks before baseline, and headache medication prescribed in the 26 weeks before baseline *and* in use at baseline. In addition, medication overuse, as assessed by the treating physician (not required to be based on ICHD criteria), is also recorded. All headache medications and therapies are categorised by indication (ie, acute, preventative, complementary) and drug class, and any changes in medication use are documented at each follow-up visit.

#### OnabotulinumtoxinA utilisation

Details of injection dose and site are documented during each treatment visit. For each visit, treating physicians document the total dose per treatment session and dose per muscle injected, as well as the number of muscle areas injected and the total number and locations of injection sites. These data are recorded for all patients receiving the PREEMPT recommended basic injections as well as for those patients who are treated with the ‘follow-the-pain’ strategy.

#### Safety and tolerability

The safety of onabotulinumtoxinA treatment is assessed via adverse drug reaction (ADR) reporting, including frequency, severity (ie, mild, moderate, severe), and relation (ie, definite, probable, possible, not assessable) to onabotulinumtoxinA. All treating physicians are required to document ADRs in the eCRF. Physician- and patient-reported onabotulinumtoxinA tolerability is rated as ‘insufficient,’ ‘moderate,’ ‘good,’ or ‘very good,’ and reported as the proportion of patients and physicians who rate the tolerability to be ‘good’ or ‘very good.’

### Statistical analysis

All baseline, demographic, efficacy, and safety analyses are performed in the safety analysis set (ie, all patients who receive ≥1 dose of onabotulinumtoxinA). Descriptive statistics are used to describe continuous variables; frequency and proportion distributions are used to report categorical data. Changes from baseline at each follow-up visit are analysed at the two-sided 5% level using a nonparametric Wilcoxon signed rank test. All data are analysed as reported in the database using SAS® version 9.3 (SAS Institute, Inc., Cary, NC); any missing data are reported as such. If any dimension score on the EQ-5D or question on the MSQ is missing, the corresponding dimension and total scores are reported as missing.

## Results

### Disposition and demographics

**Fig. 1:**
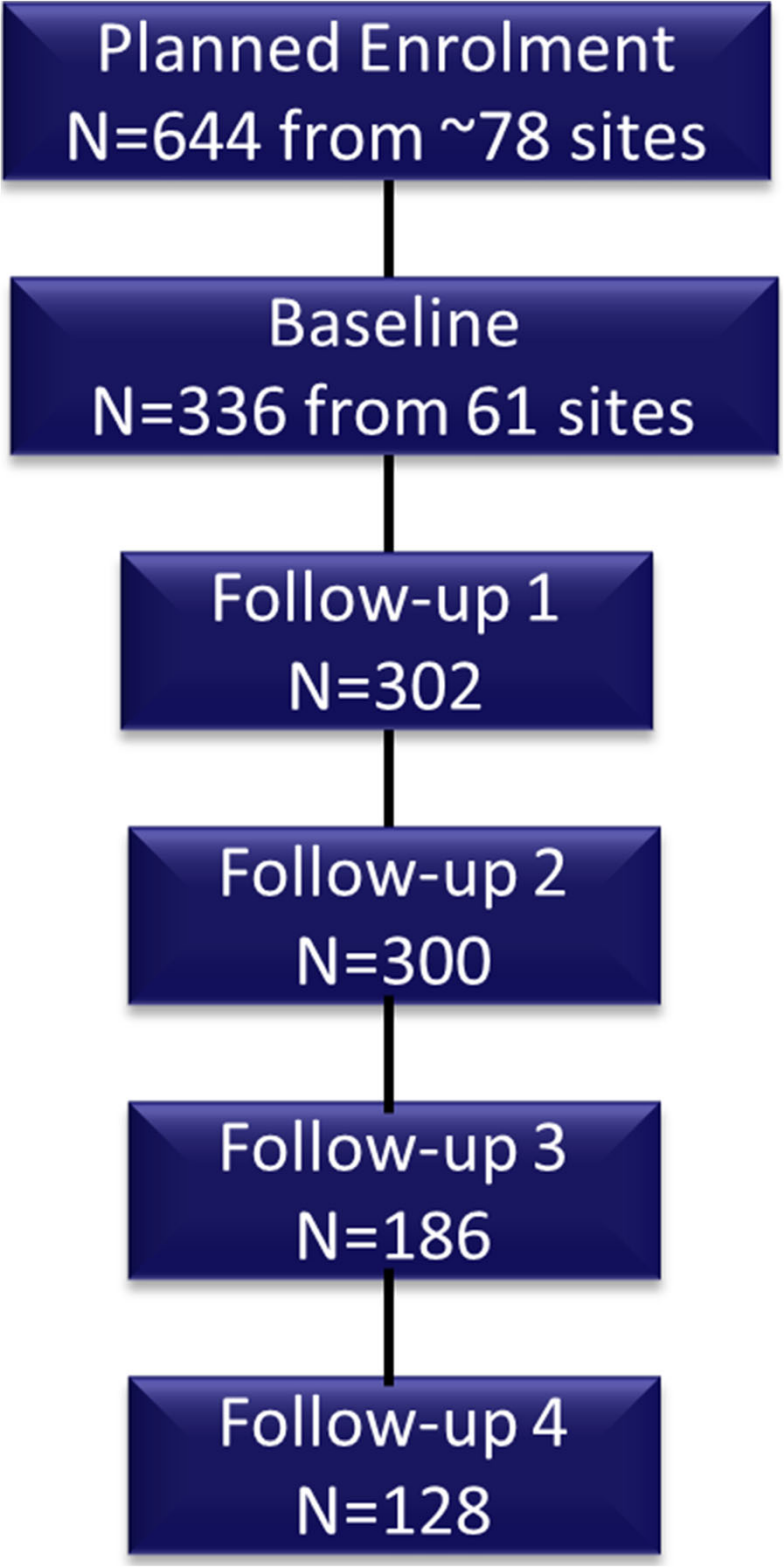
Patient Disposition

Since the first patient was enrolled in July, 2012, the study has enrolled 644 patients from 78 sites throughout Europe. As of the cutoff date for this interim analysis (February 12, 2015), baseline data are available from 61 sites for 336 patients who participated in the study for ≥6 months. All patients received ≥1 dose of onabotulinumtoxinA and are included in the safety analysis set. The number of patients who completed and provided data for baseline and up to 4 follow-up visits is displayed in Fig. 1. Four patients discontinued the study (no future treatments scheduled, *n* = 2; discontinued onabotulinumtoxinA, *n* = 2); no specific reasons for discontinuing onabotulinumtoxinA were specified in the eCRF.

Baseline patient demographic characteristics are presented in Table 2. The mean (SD) age was 44.9 (11.4) years and BMI was 24.5 (4.5) kg/m^2^. Most patients (85.4%) were women, had a secondary school certificate (55.1%), and were employed either full- or part-time (66.1%).

**Table 2:** Patient Demographics

Characteristic	6-Month Completers (*n* = 336) ^a^
Age (years)	
Mean (SD)	44.9 (11.4)
Median (min, max)	46.0 (18, 76)
Age group (years), n (%)	
≥ 18 and <30	33 (9.8)
≥ 30 and <40	80 (23.8)
≥ 40 and <50	107 (31.8)
≥ 50	115 (34.2)
Gender, n (%)	
Male	49 (14.6)
Female	287 (85.4)
Weight (kg)	
Mean (SD)	68.6 (14.3)
Median (min, max)	65 (46, 150)
Height (cm)	
Mean (SD)	167.4 (8.3)
Median (min, max)	167 (150, 203)
Body mass index (kg/m^2^)	
Mean (SD)	24.5 (4.5)
Median (min, max)	24 (16, 43)
Education, n (%)^b^	
No school-leaving qualifications	6 (1.8)
Still attending school	0
Secondary school certificate (‘Hauptschule’)	69 (20.5)
Secondary school certificate (‘Realschule’)	116 (34.5)
Higher education entrance qualification	71 (21.1)
University degree	59 (17.6)
Employment Status, n (%)^c^	
Full time	179 (53.3)
Part time	43 (12.8)
Retiree	20 (6.0)
Unemployed	20 (6.0)
Pupil	1 (0.3)
Trainee	3 (0.9)
Student	6 (1.8)
Self-employed	10 (3.0)
Housewife/husband	26 (7.7)
Side job	4 (1.2)
Pensioner	15 (4.5)

^a^Only gender data was available for 1 patient

^b^Education data were unavailable for 15 patients

^c^Employment data were unavailable for 9 patients

### Medical and headache history

Patient headache-related disease history at baseline is presented in Table 3. The mean (SD) patient-reported age of headache onset was 18.5 (9.3) years. The mean (SD) time since migraine diagnosis was 19.5 (12.1) years and CM diagnosis was 5.1 (7.6) years. The mean (SD) patient-estimated headache-day frequency (ie, any day with ≥4 h of continuous headache) was 20.6 (5.3) days per month. Based on the treating physician’s clinical judgment, it was determined that approximately one-third of the patients (34.2%) had CM complicated by medication overuse (including medication overuse, rebound, or analgesic overuse headaches). Baseline quality of life measures indicate substantial disease burden, as measured by mean (SD) MSQ (56.2 [12.4]), EQ-5D index (0.47 [0.38]), and EQ-5D VAS scores (47.7 [25.1]).

**Table 3:** Disease History of Patients with CM

		6-Month Completers (*n* = 336)
Time since first diagnosis of CM, years	N	336
	Mean (SD)	5.1 (7.6)
	Median (min, max)	1.8 (−0.8, 40.6)^a^
Age of onset, years^b^	N	331
	Mean (SD)	18.5 (9.3)
	Median (min, max)	16 (3, 57)
Time since first diagnosis of migraine, years	N	330
	Mean (SD)	19.5 (12.1)
	Median (min, max)	19.8 (−0.2, 71)^a^
Non-CM headache diagnoses, n (%)^d^	Migraine	245 (72.9)
	Medication overuse^c^	115 (34.2)
	Tension headache	83 (24.7)
	Chronic tension-type headache	41 (12.2)
	Chronic daily headache	39 (11.6)
	Menstrual headache or menstrual migraine	28 (8.3)
	Stress headache	28 (8.3)
	Intractable/refractory migraine or headache	21 (6.3)
	Cluster headache	8 (2.4)
	Sinus headache	4 (1.2)
	Hemicrania continua	1 (0.3)
	New daily persistent headache	0
	Other	5 (1.5)

CM = chronic migraine

^a^The negative minimum value results from incorrect dates in the electronic case report form. The date of the baseline visit was prior to the date of the first diagnosis in 1 patient

^b^Based on the patient’s recollection

^c^Includes any diagnosis of medication overuse, rebound, or analgesic overuse headache, based on the treating physician’s clinical judgment

^d^Multiple answers were possible

### Healthcare resource and medication utilisation

In the 3 months before baseline, a small proportion (~4%) of patients had been admitted to the hospital for headache. Conversely, HCP visits were more frequent; 42.9% of patients had a mean (SD) of 3.5 (4.0) HCP visits in the 3 months before baseline. The most frequently used (ie, ≥50% of patients) headache medications at any time before the study were beta blockers, antidepressants, and antiepileptics (~70% each). Only 7.1% of patients had previously received botulinum toxin for treatment of headache (Table 4). During the 26 weeks before baseline, a large proportion (90.8%) of patients had been prescribed some type of headache medication. At baseline, 80.7% of patients were using prescribed acute headache medications, resulting in approximately one-fifth of patients (19.3%) not using prescribed acute headache medications. Prescribed preventative headache medications were in use by 50.0% of the patients. The most frequently used (prescription and nonprescription) acute headache medications were triptans and ibuprofen, and the most frequent preventative medication was topiramate (Table 4). According to the treating physician, ~40% of patients were overusing their headache medications at baseline.

**Table 4:** Medication History

	6-Month Completers (*n* = 336)
Previous headache medication, n (%)^a^	
Beta blockers	238 (70.8)
Antidepressants	235 (69.9)
Antiepileptics	227 (67.6)
Calcium channel blockers	102 (30.4)
Botulinum toxin	24 (7.1)
Medication/therapy prescribed in the 26 weeks before baseline, n (%)^a^	
Any headache medication/therapy	305 (90.8)
Acute treatment for headache	283 (84.2)
Headache prevention	211 (62.8)
Complementary therapies	73 (21.7)
Medication/therapy prescribed in the 26 weeks before baseline and used by the patient at baseline, n (%) ^a,b^	
Headache prevention	168 (50.0)
Topiramate	57 (17.0)
Acute treatment for headache	271 (80.7)
Sumatriptan	95 (28.3)
Ibuprofen	75 (22.3)
Rizatriptan	43 (12.8)
Zolmitriptan	40 (11.9)
Complementary therapies	49 (14.6)

^a^Multiple answers were possible

^b^Medications/therapies which were used at baseline by at least 10% of the patients

## Discussion

To date, the PREEMPT clinical trial programme is the largest study of onabotulinumtoxinA in CM and provides evidence for safety and efficacy over a 52-week treatment period [23–25]. The purpose of the REPOSE study is to provide real-world data, observed in a clinical setting, regarding the use of onabotulinumtoxinA for the treatment of CM over a 2-year period, as measured by patient- and physician-reported efficacy outcomes, healthcare resource utilisation, and adverse event profile. These data are intended to complement and strengthen previously published rigorously controlled studies and real-world analyses by supplying previously unreported information about the real-world long-term impact of onabotulinumtoxinA on CM patient healthcare utilisation patterns and disease burden including poor quality of life, which is an important distinguishing feature of CM [39, 40].

As of the database cutoff date, baseline data were available for approximately half (336/644) of the enrolled patient population. Consistent with previous research [10, 41, 7], the majority of patients (~80%) in the current study are female, with a mean age of ~45 years, substantial disease burden (as measured by the MSQ), and most have previously used beta-blockers, antidepressants, or antiepileptics for treatment of CM. Nevertheless, some differences were observed between this European population and the US population of patients with CM. One notable difference is the mean BMI; patients in the current study have a lower BMI (24.5 kg/m^2^) than that observed among patients with CM in a large US epidemiologic study (American Migraine Prevalence and Prevention Study [AMPP]; 30.4 kg/m^2^) [6]. This dissimilarity may reflect international differences in BMI (general population mean BMI: 23.8 to 25.3 kg/m^2^ for women and 25.5 to 26.3 kg/m^2^ for men in 9 European countries [42]; 28.7 kg/m^2^ for women and 28.6 kg/m^2^ for men in the United States [43]. Additionally, patients in the current study have higher rates of full- or part-time employment (66.1%) compared with those from 2 US epidemiologic studies (Chronic Migraine Epidemiology and Outcomes Study, 56.4%; AMPP, 47%) [44, 45], although, again, this may partly reflect population differences due to study design.

In contrast with expectations, an unexpected low rate of intractable/refractory migraine or headache was observed, which may be attributed to inconsistencies in diagnosis among treating physicians or a possible low participation of headache specialists in the study. Coinciding with the timeframe of this study, there was debate on the definition of refractory CM (due to the introduction of onabotulinumtoxinA as a preventive treatment), resulting in a proposal from the European Headache Federation Expert Group to modify the definition to also include patients who had contraindications to or no effect from onabotulinumtoxinA treatment. [46] This “tightening” of the definition of refractory CM could have led to the low rate of intractable/refractory migraine observed. In addition, the time since first diagnosis of migraine and CM were ~20 and ~5 years, respectively, which may indicate that migraine chronification occurs over a protracted period. Although adherence is not being directly measured in the current study, approximately one-fifth of patients are not using their acute headache medications as prescribed. Furthermore only 50% of patients are using their prescribed preventative medications at baseline. This is slightly higher than the ~30% adherence rate observed in a study of the adherence of preventative oral medications for CM [18]; however, further investigation is required to understand why patients with CM are not taking their medications as prescribed, but it is possible that a lack of sustained effectiveness or the presence of troublesome side effects contribute to poor compliance.

This report of baseline data from the REPOSE study has some limitations. The observational nature of the study reflecting real-world use of onabotulinumtoxinA precludes any utilisation of formal protocol requirements or exclusion criteria. One consequence of this study design is that treatments were not mandated, but were provided at the participating physicians’ discretion according to their clinical judgment and local standards of medical care. Another consequence is the observed variety of diagnoses and the inability to disentangle comorbidities that might influence treatment outcomes. In addition, since headache diaries were not used, the patient-reported outcomes relied on patient recollection and reporting compliance, which may result in incomplete and/or missing data.

## Conclusion

The REPOSE Study will provide the largest real-world, long-term analysis of the clinical use of onabotulinumtoxinA for the treatment of CM and will add important information to existing real-world findings. Future analyses will assess the long-term safety and efficacy of onabotulinumtoxinA in this population.

## Abbreviations

ADR: Adverse drug reaction
AE: Adverse event
AMPP: American Migraine Prevalence and Prevention Study
BMI: Body mass index
CM: Chronic migraine
CM-PASS: Botox Chronic Migraine Post-Authorisation Safety Study
eCRF: Electronic case report form
EM: Episodic migraine
EQ-5D: EuroQol Group Questionnaire
HCP: Healthcare professional
ICHD-3b: International Classification of Headache Disorders (third edition, beta version)
MSQ: Migraine-Specific Quality of Life Questionnaire
NA: Not applicable
PREEMPT: The Phase 3 REsearch Evaluating Migraine Prophylaxis Therapy
REPOSE: REal-life use of botulinum toxin for the symptomatic treatment of adults with chronic migraine, measuring healthcare resource utilisation, and Patient-reported OutcomeS observed in practice
VAS: Visual analog scale

## References

[1] Centers for Disease Control and Prevention (2014) Summary Health Statistics for U.S. Adults: National Health Interview Survey, 2012

[2] Burch RC, Loder S, Loder E, Smitherman TA (2015). The prevalence and burden of migraine and severe headache in the United States: updated statistics from government health surveillance studies. Headache.

[3] Stovner LJ, Andree C (2010). Prevalence of headache in Europe: a review for the Eurolight project. J headache pain.

[4] Headache Classification Committee of the International Headache Society (2013). The international classification of headache disorders, 3rd edition (beta version). Cephalalgia.

[5] Natoli JL, Manack A, Dean B, Butler Q, Turkel CC, Stovner L, Lipton RB (2010). Global prevalence of chronic migraine: a systematic review. Cephalalgia.

[6] Buse DC, Manack AN, Serrano D, Reed ML, Varon S, Turkel CC, Lipton RB (2012). Headache impact of chronic and episodic migraine: results from the American migraine prevalence and prevention study. Headache.

[7] Blumenfeld AM, Varon SF, Wilcox TK, Buse DC, Kawata AK, Manack A, Goadsby PJ, Lipton RB (2011) Disability, HRQoL and resource use among chronic and episodic migraineurs: results from the International Burden of Migraine Study (IBMS). Cephalalgia 31:301–315. doi:10.1177/0333102410381145 10.1177/0333102410381145 20813784

[8] Lanteri-Minet M, Duru G, Mudge M, Cottrell S (2011). Quality of life impairment, disability and economic burden associated with chronic daily headache, focusing on chronic migraine with or without medication overuse: a systematic review. Cephalalgia.

[9] Ruscheweyh R, Muller M, Blum B, Straube A (2014). Correlation of headache frequency and psychosocial impairment in migraine: a cross-sectional study. Headache.

[10] Bloudek LM, Stokes M, Buse DC, Wilcox TK, Lipton RB, Goadsby PJ, Varon SF, Blumenfeld AM, Katsarava Z, Pascual J, Lanteri-Minet M, Cortelli P, Martelletti P (2012) Cost of healthcare for patients with migraine in five European countries: results from the International Burden of Migraine Study (IBMS). J headache pain 13:361–378. doi:10.1007/s10194-012-0460-7 10.1007/s10194-012-0460-7 PMC3381065 22644214

[11] Munakata J, Hazard E, Serrano D, Klingman D, Rupnow MF, Tierce J, Reed M, Lipton RB (2009) Economic burden of transformed migraine: results from the American Migraine Prevalence and Prevention (AMPP) study. Headache 49:498–508. doi:10.1111/j.1526-4610.2009.01369.x 10.1111/j.1526-4610.2009.01369.x 19245386

[12] Rothrock JF, Bloudek LM, Houle TT, Andress-Rothrock D, Varon SF (2014) Real-world economic impact of onabotulinumtoxinA in patients with chronic migraine. Headache 54:1565–1573. doi:10.1111/head.12456 10.1111/head.12456 PMC4282490 25298117

[13] Starling AJ, Dodick DW (2015). Best practices for patients with chronic migraine: burden, diagnosis, and management in primary care. Mayo Clin Proc.

[14] Silberstein SD, Lipton RB, Dodick DW, Freitag FG, Ramadan N, Mathew N, Brandes JL, Bigal M, Saper J, Ascher S, Jordan DM, Greenberg SJ, Hulihan J (2007). Efficacy and safety of topiramate for the treatment of chronic migraine: a randomized, double-blind, placebo-controlled trial. Headache.

[15] Diener HC, Bussone G, Van Oene JC, Lahaye M, Schwalen S, Goadsby PJ (2007). Topiramate reduces headache days in chronic migraine: a randomized, double-blind, placebo-controlled study. Cephalalgia.

[16] Santiago MD, Carvalho Dde S, Gabbai AA, Pinto MM, Moutran AR, Villa TR (2014). Amitriptyline and aerobic exercise or amitriptyline alone in the treatment of chronic migraine: a randomized comparative study. Arq Neuropsiquiatr.

[17] Couch JR, Amitriptyline Versus Placebo Study G (2011). Amitriptyline in the prophylactic treatment of migraine and chronic daily headache. Headache.

[18] Hepp Z, Dodick DW, Varon SF, Gillard P, Hansen RN, Devine EB (2015). Adherence to oral migraine-preventive medications among patients with chronic migraine. Cephalalgia.

[19] Hepp Z, Bloudek LM, Varon SF (2014). Systematic review of migraine prophylaxis adherence and persistence. J Manag Care Pharm.

[20] Serrano D, Buse DC, Manack Adams A, Reed ML, Lipton RB (2015) Acute treatment optimization in episodic and chronic migraine: results of the American Migraine Prevalence and Prevention (AMPP) study. Headache 55:502–518 10.1111/head.12553 25881676

[21] Allergan, Inc. (2015) Summary of product characteristics (Botox® [onabotulinumtoxinA for injection])

[22] BOTOX® COSMETIC (onabotulinumtoxinA) for injection, for intramuscular use. Full Prescribing Information, Allergan, Inc., Irvine, CA, 2013

[23] Aurora SK, Dodick DW, Turkel CC, DeGryse RE, Silberstein SD, Lipton RB, Diener HC, Brin MF (2010). OnabotulinumtoxinA for treatment of chronic migraine: results from the double-blind, randomized, placebo-controlled phase of the PREEMPT 1 trial. Cephalalgia.

[24] Diener HC, Dodick DW, Aurora SK, Turkel CC, DeGryse RE, Lipton RB, Silberstein SD, Brin MF (2010). OnabotulinumtoxinA for treatment of chronic migraine: results from the double-blind, randomized, placebo-controlled phase of the PREEMPT 2 trial. Cephalalgia.

[25] Aurora SK, Winner P, Freeman MC, Spierings EL, Heiring JO, DeGryse RE, VanDenburgh AM, Nolan ME, Turkel CC (2011). OnabotulinumtoxinA for treatment of chronic migraine: pooled analyses of the 56-week PREEMPT clinical program. Headache.

[26] Ahmed F, Zafar HW, Buture A, Khalil M (2015) Does analgesic overuse matter? Response to OnabotulinumtoxinA in patients with chronic migraine with or without medication overuse. SpringerPlus 4:589. doi:10.1186/s40064-015-1386-8 10.1186/s40064-015-1386-8 PMC4628076 26543724

[27] Negro A, Curto M, Lionetto L, Crialesi D, Martelletti P (2015). OnabotulinumtoxinA 155 U in medication overuse headache: a two years prospective study. Spring.

[28] Negro A, Curto M, Lionetto L, Martelletti P (2015). A two years open-label prospective study of OnabotulinumtoxinA 195 U in medication overuse headache: a real-world experience. J Headache Pain.

[29] Pedraza MI, de la Cruz C, Ruiz M, Lopez-Mesonero L, Martinez E, de Lera M, Guerrero AL (2015). OnabotulinumtoxinA treatment for chronic migraine: experience in 52 patients treated with the PREEMPT paradigm. Spring.

[30] Khalil M, Zafar HW, Quarshie V, Ahmed F (2014) Prospective analysis of the use of OnabotulinumtoxinA (BOTOX) in the treatment of chronic migraine; real-life data in 254 patients from Hull, U.K. J Headache Pain 15:54 10.1186/1129-2377-15-54 PMC4166400 25178393

[31] Guerzoni S, Pellesi L, Baraldi C, Pini LA (2015). Increased efficacy of regularly repeated cycles with OnabotulinumtoxinA in MOH patients beyond the first year of treatment. J Headache Pain.

[32] Grazzi L, Usai S (2015) Onabotulinum toxin A (Botox) for chronic migraine treatment: an Italian experience. Neurol Sci 36(Suppl 1):33–35 10.1007/s10072-015-2140-2 26017508

[33] Kollewe K, Escher CM, Wulff DU, Fathi D, Paracka L, Mohammadi B, Karst M, Dressler D (2016). Long-term treatment of chronic migraine with OnabotulinumtoxinA: efficacy, quality of life and tolerability in a real-life setting. J Neural Transm (Vienna).

[34] Allergan plc Botox® (onabotulinumtoxinA for injection). Summary of Product Characteristics, Allergan plc, Irvine, CA, USA (2015). Botox® (onabotulinumtoxinA for injection). Summary of Product Characteristics.

[35] Blumenfeld A, Silberstein SD, Dodick DW, Aurora SK, Turkel CC, Binder WJ (2010). Method of injection of onabotulinumtoxinA for chronic migraine: a safe, well-tolerated, and effective treatment paradigm based on the PREEMPT clinical program. Headache.

[36] Martin BC, Pathak DS, Sharfman MI, Adelman JU, Taylor F, Kwong WJ, Jhingran P (2000). Validity and reliability of the migraine-specific quality of life questionnaire (MSQ version 2.1). Headache.

[37] Cole JC, Lin P, Rupnow MF (2007) Validation of the Migraine-Specific Quality of Life Questionnaire version 2.1 (MSQ v. 2.1) for patients undergoing prophylactic migraine treatment. Qual Life Res 16:1231–1237. doi:10.1007/s11136-007-9217-1 10.1007/s11136-007-9217-1 17468941

[38] Walters SJ, Brazier JE (2005). Comparison of the minimally important difference for two health state utility measures: EQ-5D and SF-6D. Qual Life Res.

[39] Meletiche DM, Lofland JH, Young WB (2001). Quality-of-life differences between patients with episodic and transformed migraine. Headache.

[40] Wang SJ, Wang PJ, Fuh JL, Peng KP, Ng K (2012) Comparisons of disability, quality of life, and resource use between chronic and episodic migraineurs: a clinic-based study in Taiwan. Cephalalgia. doi:10.1177/0333102412468668 10.1177/0333102412468668 23203506

[41] Katsarava Z, Buse DC, Manack AN, Lipton RB (2012) Defining the differences between episodic migraine and chronic migraine. Curr Pain Headache Rep 16:86-92. doi:10.1007/s11916-011-0233-z 10.1007/s11916-011-0233-z PMC3258393 22083262

[42] Garcia Villar J, Quintana-Domeque C (2009). Income and body mass index in Europe. Econ Hum Biol.

[43] Fryar CD, Gu Q, Ogden CL (2012) Anthropometric reference data for children and adults: United States, 2007-2010. Vital Health Stat 111-48 25204692

[44] Manack Adams A, Serrano D, Buse DC, Reed ML, Marske V, Fanning KM, Lipton RB (2015) The impact of chronic migraine: the Chronic Migraine Epidemiology and Outcomes (CaMEO) Study methods and baseline results. Cephalalgia 35:563–578. doi:10.1177/0333102414552532 10.1177/0333102414552532 PMC4430584 25304766

[45] Stewart WF, Wood GC, Manack A, Varon SF, Buse DC, Lipton RB (2010) Employment and work impact of chronic migraine and episodic migraine. J Occup Environ Med 52:8–14. doi:10.1097/JOM.0b013e3181c1dc56 10.1097/JOM.0b013e3181c1dc56 20042889

[46] Martelletti P, Katsarava Z, Lampl C, Magis D, Bendtsen L, Negro A, Russell MB, Mitsikostas DD, Jensen RH (2014) Refractory chronic migraine: a consensus statement on clinical definition from the European Headache Federation. J Headache Pain 15:47 10.1186/1129-2377-15-47 PMC4237793 25169882

